# Life after the hospital: Lived experience of people with traumatic spinal cord injury in a resource-constrained setting – a qualitative study

**DOI:** 10.4102/sajp.v82i1.2273

**Published:** 2026-02-20

**Authors:** Maurice Kanyoni, Margaret I. Fitch, Joliana Phillips, Lena Nilsson-Wikmar, David K. Tumusiime

**Affiliations:** 1 Department of Physiotherapy, School of Health Sciences, University of Rwanda, Kigali, Rwanda; 2 Lawrence Bloomberg Faculty of Nursing, University of Toronto, Toronto, Canada; 3 Department of Physiotherapy, School of Health Sciences, University of the Western Cape, Cape Town, South Africa; 4 Department of Neurobiology, Care Sciences and Society, Division of Physiotherapy, Karolinska Institutet, Stockholm, Sweden

**Keywords:** lived experience, traumatic spinal cord injury, qualitative study, Rwanda and East Africa

## Abstract

**Background:**

When a traumatic spinal cord injury (TSCI) happens to healthy individuals, it requires an adjustment to life situations in the community. Exploring the lived experience of spinal cord injury survivors is important because it forms a foundation for designing strategies to improve their reintegration back into the community. There are limited studies in the East and Central African region regarding the experience of community reintegration following a TSCI.

**Objectives:**

This study explored lived experiences of persons with TSCI in Rwanda. Specifically, it sought to identify and understand the barriers and facilitators to living in the community following a TSCI.

**Method:**

A descriptive qualitative design was employed. Nineteen individuals, purposively selected for diversity, were interviewed face to face. Interviews were audio-recorded and transcribed. Data were thematically analysed.

**Results:**

The mean age of the informants was 40 years, ranging from 21 years to 72 years old. Ten participants had paraplegia, and nine were living with tetraplegia. The themes identified were personal factors, social relationships, community-related factors, preinjury status, and having common conditions. Strong personal resources, a supportive family, health insurance coverage, and peer counselling were reported as facilitators. Barriers include inaccessibility to public buildings and transport, inappropriate assistive devices, inappropriate language and pre-injury conditions.

**Conclusion:**

The challenges experienced by people with TSCI range from personal to environmental factors, and from employment to policy issues. This study sheds light on the lived experience of individuals with TSCI. There is a need to review current relevant policies in Rwanda as a first step to addressing these issues.

**Clinical implications:**

Rehabilitation services in Rwanda need to be designed to include home-based care. Introducing peer counselling could be beneficial within the rehabilitation programme.

## Introduction

Traumatic spinal cord injury (TSCI) is a sudden life-changing condition that affects every dimension of life, including the physical, social, and psychological wellbeing of individuals (Fuseini, Aniteye & Alhassan 2019). The sudden occurrence of TSCI can be overwhelming and have a critical impact on the individual and significant others (Babamohamadi, Negarandeh & Dehghan-Nayeri 2011). Significant improvements in survival rates have been reported, particularly in high-income countries because of improved health and social welfare, whilst limited access and poorly equipped health care services in low-income countries lead to low survival rates (Cripps et al. 2011; World Health Organization [WHO] 2013). Higher survival rates for patients with TSCI will lead to the likelihood of living with a disability for longer periods of time and experiencing additional costs for families, leading to stress amongst individuals with spinal cord injury (SCI) (WHO 2013). Yet, like many persons with disabilities, those with SCI continue to experience substantial health inequities. According to the Convention on the Rights of Persons with Disabilities (CRPD), member states must ensure that persons with SCI can access the same range, quality, and standard of free or affordable health care and social support as others; thus, addressing inequities is essential (United Nations; WHO 2023). Rwanda signed and ratified the CRPD in 2008.

Most rehabilitation professionals see people with SCI in clinical settings and may not have an understanding of the social context of this specific disability for the individual (Wong et al. 2022). The short period of hospital stays and contact with healthcare professionals soon after the injury makes it difficult for staff to understand environmental conditions surrounding people with SCI after discharge. Arguably, the ultimate goal of rehabilitation post-SCI is optimal community reintegration (Akter et al. 2019). Successful community reintegration of people with SCI requires patients and their families to adapt to changes with respect to the physical and psychosocial functioning after injury (Babamohamadi et al. 2011).

Studies have been conducted in high- and middle-income countries, but only a limited number of studies exist from low-income countries, particularly in Africa, and most of these represent a small region of a country, like a single district or town-level experience. For example, a study in Tamale, Ghana, regarding the lived experience of individuals with SCI (Babamohamadi et al. 2011; Fuseini et al. 2019; WHO 2003) reported that physical and physiological impairments affect individuals with SCI socially and psychologically and reduce the overall quality of life. In Rwanda, the challenges of living at home following a traumatic injury have not been systematically explored and documented. Such exploration would be valuable to determine the experiences of functioning and wellbeing of individuals with SCI.

In Rwanda, there are no specialised SCI rehabilitation services other than four tertiary-level acute hospitals providing spinal surgery and one rehabilitation centre with an SCI rehabilitation unit where patients receive occupational therapy, physiotherapy, and psychotherapy. There is no national registry of TSCI, but a 2020 epidemiology study estimates there are 22.2 per million people with TSCI (Kanyoni et al. 2024a). This study was followed by an evaluation of psychosocial reintegration (Kanyoni et al. 2024b) and quality of life (Kanyoni et al. 2023), which showed the results for both were poor; for example, the only available housing arrangements for individuals following a TSCI are their family household. An in-depth understanding of the enabling and disabling contexts of people with TSCI in the community would inform rehabilitation systems and practice and national policies. Based on this background, this study used a descriptive qualitative approach to explore the lived experiences of persons living with TSCI in Rwanda.

## Research methods and design

### Design and sample

This study utilised a descriptive qualitative design, as this methodology provides an in-depth overview of a particular phenomenon. To achieve a broad understanding of the experiences of people living with TSCI, typical case purposive sampling was used to recruit participants. This study adopted a constructionist ontology, viewing the reality as socially constructed. Epistemologically, the study is based on an interpretivist approach, aiming to understand participants’ subjective experiences.

Knowledge is co-created through the interaction between researcher and participants. The authors are experienced researchers in the field of TSCI and have vast experience in the rehabilitation of TSCI. Our background and perspective influenced both data collection and interpretation, rather than claiming neutrality.

This qualitative study was part of a large incident study that was conducted in Rwanda between October 2019 and September 2020 (Kanyoni et al. 2024a). The incident study resulted in a database, which included all individuals who suffered a TSCI during the period (*N* = 122). For this qualitative study, we interviewed participants until we reached data saturation, when no new information was appearing, which occurred at the 19th interview.

Participants were selected based on the following criteria: (1) living with a TSCI for at least 1 year, (2) an adult survivor 18 years and above, (3) made a transition from the hospital setting to community life, and (4) were willing and able to describe their experiences. Informants were also selected to reflect diversity criteria of gender, age, marital status, level of injury, and place of residence (rural vs. urban). The criteria were seen as characteristics that may influence the individuals’ experience in the community. Interviews were conducted from 01 July 2022 up to 15 August 2022.

### Study setting

The study was carried out in Rwanda, one of the smallest countries on the African continent. According to the last census of 2012, the Rwandan population is estimated at 10 515 973 people, spread across an area of 26 340 km^2^ with 5 500 845 who are aged 18 years and above (National Institute of Statistics of Rwanda and Ministry of Finance and Economic Planning 2014).

Participants in this study were spread throughout the country, living in their respective households in towns and rural areas; therefore, the study was conducted in the whole country.

### Data collection

The first author (Maurice Kanyoni) visited with the eligible participants prior to actual data collection or interviews to explain the purpose of the study and procedures. Potential participants were informed there were no right or wrong answers in the interviews and they could withdraw at any time without penalties (Shenton 2004). Potential participants were able to ask any questions about the study, and all who agreed to participate provided written consent prior to the interviews being conducted.

Face-to-face, individual interviews were conducted on a second visit with the participants by the first author (Maurice Kanyoni), a physical therapist with more than 10 years of experience with inpatient and community rehabilitation, including SCI rehabilitation. The authors were completely unknown to the participants.

The interview explored the experiences of participants before and after their injury using an interview guide, a guide specifically developed for this study by all authors except the second author. The open-ended questions were framed broadly to allow the individuals to speak about the topics that were important to them. It comprised the following broad questions: (1) Describe your life experience before the injury. (2) In what ways has the injury had an effect on your life? (3) Consider negative and positive aspects. (4) How do you manage your daily life?

(5) Can you describe the role of your family in your daily life? (6) Can you describe the challenges and obstacles that you face in your daily life? (7) How do you experience attitudes from your family and society? Probes were used to enrich the discussion when necessary. The interviews were conducted in *Kinyarwanda*.

Depending on the informants’ availability, 14 participants were interviewed at their homes and five were interviewed at the rehabilitation centre. All 19 interviews were audio recorded and later transcribed verbatim into *Kinyarwanda*. The shortest was 32 min, whilst the longest was 68 min.

### Data management and analysis

The first two audio-recorded interviews were translated verbatim into English by the first author (Maurice Kanyoni) and shared with the second author (Margaret I. Fitch), who had expertise in rehabilitation and qualitative data analysis. The two discussed the aim of the study and agreed on the definition of experiences and its attributes. Each read the transcripts several times and coded independently based on the discussion about experiences living with TSCI and its attributes. The two compared their codes and agreed upon the final coding framework.

**FIGURE 1:**
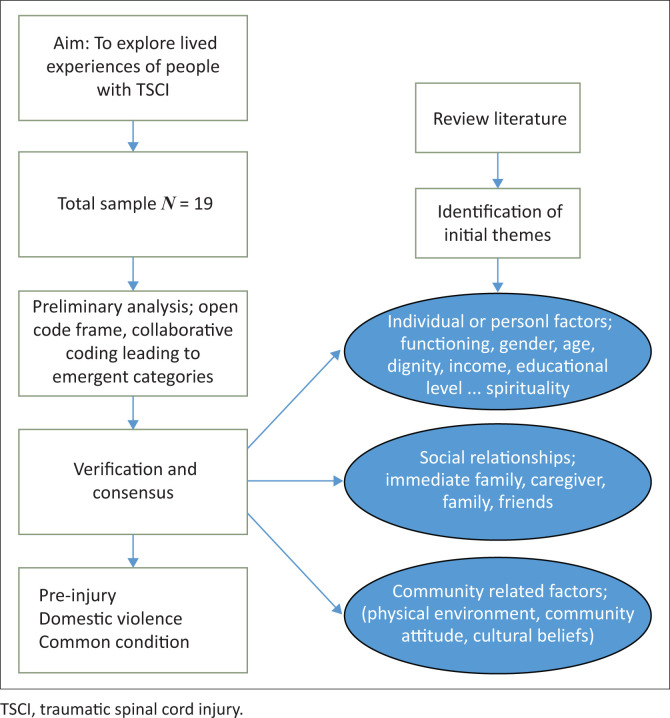
Data collection and analysis.

The analysis utilised a deductive-inductive approach (Figure 1). Based on the literature regarding experiences of SCI survivors living in the community (Donovan 2007; Fuseini et al. 2019; Joseph et al. 2016; Löfvenmark et al. 2016; WHO 2013), three broad topic areas were identified as beginning themes for analysis: individual or personal factors, social relationships, and community-related factors (Akter et al. 2019; Hisham et al. 2022).

Individual/personal-related factors are those that are linked to functioning (e.g., impairments, abilities, and limitations), age category, income levels, spirituality, and gender. The social relationships category concerned immediate family support, which could be spouse/partner, parent, sibling, caregiver, friend, and workmate support. The third category included those factors that are community related, such as physical environment accessibility, community attitudes, and cultural beliefs. These broad topic areas/themes, drawn from existing literature, were used to classify the study participants’ experiences, applying a deductive synthesis approach to the data (Nowell et al. 2017). However, in the process of coding the data, ideas were identified which did not fit in with the predetermined topics. Hence new topic areas were added, including preinjury factors (i.e. teenage pregnancy and financial hardships) and having a condition in common. Discussion and consensus regarding the results occurred between the two researchers (Maurice Kanyoni and Margaret I. Fitch). Authors Maurice Kanyoni, Lena Nilsson-Wikmar and David K. Tumusiime checked several times for similarities and any difference in the code frames generated vis-à-vis the previously agreed coding frame.

Following the determination of the coding framework, all remaining interview transcripts were analysed by Maurice Kanyoni in *Kinyarwanda*. Simultaneous data collection and analysis helped to determine saturation. The interview data were transferred to a qualitative analysis tool called ‘ATLAS.ti’, software that stores, organises, and retrieves data in text form. The software enables collaborative coding, grouping of codes, and the development of categories.

### Methodological rigour

Trustworthiness of the analysis was improved by considering credibility, transferability, dependability, and confirmability during the whole process (Shenton 2004). Two research experts in qualitative methods constantly reviewed transcripts and data collection progress and provided feedback. The transcripts were read many times by the first (Maurice Kanyoni) and second author (Margaret I. Fitch), and data collection and analysis were carried out simultaneously to allow any possible change of methodology and monitor saturation (Almalki 2016). The interview guide had questions that facilitated exploration of similar experiences and community reintegration in the African context (Löfvenmark et al. 2017).

Both deductive and inductive approaches were applied in the analysis to allow capture of all aspects of participant experience (Nowell et al. 2017). To ensure dependability and confirmability (Shenton 2004), an audit trail of the conceptualisation of the study, recruitment of participants, and analyses of the data was maintained and reviewed by the senior authors.

### Ethical considerations

The study protocol was approved by the Institutional Review Board of the College of Medicine and Health Sciences at the University of Rwanda (Approval No. 308/CMHS IRB/2019) and the Rwanda National Ethics Committee (FWA Assurance No. 00001973 IRB No. 00001497). All participants signed informed consent, codes were used instead of names to maintain participant anonymity, and transcripts were only accessible by the authors on their private portable computers, which were PIN protected. The participants information sheet, consent form, and all explanations were in Kinyarwanda, a language spoken by all Rwandans; Rwanda is a monolithic nation.

## Results

### Participants’ characteristics and recruitment

A total of 19 participants were interviewed: male (*n* = 15) and female (*n* = 4). The mean age of the informants was 40 years, ranging from 21 years to 72 years. Ten participants had paraplegia, and nine were living with tetraplegia.

All were living in their family homes in the community, with 11 located in rural settings. Additional background characteristics are shown in Table 1.

**TABLE 1:** Participant demographic characteristics.

Participant code	Age (years)	Gender	Injury level	Residence setting
P1	40	Male	Paraplegia	Rural
P2	21	Female	Paraplegia	Urban
P3	34	Male	Paraplegia	Rural
P4	27	Male	Tetraplegia	Rural
P5	52	Male	Paraplegia	Rural
P6	47	Male	Tetraplegia	Urban
P7	32	Female	Paraplegia	Urban
P8	30	Male	Paraplegia	Urban
P9	45	Male	Tetraplegia	Urban
P10	35	Male	Tetraplegia	Urban
P11	72	Male	Tetraplegia	Rural
P12	67	Male	Tetraplegia	Rural
P13	28	Male	Tetraplegia	Rural
P14	63	Male	Tetraplegia	Rural
P15	35	Female	Paraplegia	Rural
P16	50	Male	Paraplegia	Rural
P17	27	Male	Paraplegia	Urban
P18	36	Male	Tetraplegia	Urban
P19	27	Female	Paraplegia	Rural

### Thematic observations

The analysis identified five themes: individual or personal factors, social relationships, community-related factors, pre-injury context and having a condition in common. The results of the deductive analysis and subcategories will be presented firstly, followed by a description of the themes and their subcategories and illustrated with participant quotes.

#### Theme 1: Individual or personal factors

The individual or personal factor’s theme describes the factors which participants perceived influenced their dignity as a person or sense of who they are now that they have a TSCI. The factors included personal resources, functional difficulties, acceptance, gender, spirituality, and complications.

**Subcategory 1.1:** Personal resources were of paramount importance for the informants to achieve a satisfactory life situation. All 19 informants reported previous self-employment or salaried or part-time employment.

However, at the time of the study, all except two (Participant 7 and Participant 9) were not earning any income and instead were dependent on their family members. Having no income not only affected their self-dignity but also their independence. Those who had an income still expressed financial concerns because of the increased living expenses when living with a disability. It was pointed out by one informant:

‘It is difficult, I lost my job even the government gives me disability pension, it is not enough for life needs. We incur a lot of expenses, for example I use pampers worth 9500frw every week, I pay a maid/helper, feed him, paying hospital bills generally life is not easy when you have SCI.’ (Participant 8)

Income insufficiency amongst TSCI affects life in all ways, from meals, housing, rehabilitation, availability of caregivers, transport, and socialisation. Not having any income was seen as the biggest challenge, and informants felt that earning income would solve most of their problems (Participant 1 – Participant 19).

**Subcategory 1.2:** Functional difficulties because of the extent of the injury were another personal factor that affected ‘dignity’. These were either because of physiological or anatomical impairments. These impairments led to limited activity and participation restrictions for the participants and affected their execution of daily tasks. Failure to execute tasks leads to participation restriction. Informants reported failure in getting employed because of functional impairments. This was frustrating, especially if that person had the task of being the breadwinner for the family, which led to a feeling of worthlessness and despair, ‘I cannot turn in bed, cannot take myself to the toilet, I no longer work. I better die because I am useless your no longer productive’ (Participant 1). Another informant (Participant 3) compared his ability before and after injury: ‘It’s painful comparing yourself before and after injury; I have tried to take my life two times’. The same informant added, ‘Its hurtful, sometimes you have to go into your house and cry, weep and come out and life continues’.

Suicidal thoughts were common during acute care; these were mentioned by Participant 8 and Participant 19. They reported feeling worthless, having fluctuating moods, depressive thoughts, and consequently feeling a need to take his or her life.

**Subcategory 1.3:** Acceptance was seen as an important personal factor. To the informants it meant ‘accepting yourself as you are’ with your limitations but being forward-thinking, focused, courageous, and seeing there is life after injury. This attitude greatly influenced self-worth, as reported by one female informant (Participant 13) with paraplegia: ‘They have to accept their situation; otherwise, they will depress … develop complications, it will be worse, forward thinking as long as your alive keep focused’. Acceptance was described as a situation of settling down and accepting living within their abilities. TSCI is an abrupt event, and acceptance takes time. Those who reported depression and non-acceptance of their condition later adapted gradually and, on average, by 6 months had identified themselves as people with disabilities with hope they could earn a living:

‘There are many things that motivates me; I did not sit, kneel, go to the toilette, I was using pampers, but now I can go the toilette, I have orthosis, I use crutches and go to the toilette, therefore given training I may go back to my salon work.’ (Participant 2)

**Subcategory 1.4:** Gender was another personal factor that impacted ‘dignity’. Whether one is male or female can influence expectations about one’s role in the family or in society. For example, being a woman with TSCI might lead you to losing your partner or, if not married, not getting married. One female informant said:

‘I have never had sexual relation since injury, no sexual feelings; I have used pampers for long, I told my husband that he should have sex but he said it is inappropriate because I will not have any feelings.’ (Participant 15)

Conversely, unmarried men reported minimal or no hope of marrying. Some said this was because of sexual dysfunction and others being looked at as physically unable. A male informant (Participant 10) who had plans of marrying his fiancé in 3 months after the injury said:

‘Of course that was the end of the plan, she came once and that was the end of the relationship, ended that very day, no call, no visit, no bye … the moment she saw that this will take long term treatment, prognosis seem poor. She decided to look for those who are fit.’ (Participant 10)

**Subcategory 1.5:** Spirituality was another personal factor described by the informants. They emphasised that when they feel no one can change the status quo, then the remaining option is to turn to God; one needs to believe and leave the rest to be attended to by God. ‘I imagine I should call God because I hear that he is the one who gives us everything because the world robbed me of my back’ (Participant 3). It is likely that following a traumatic and life-changing event, for some, this might lead to ‘injury’ to their faith, whilst, for others, the experience strengthened their faith. It was reported by participants that sometimes health professionals use spirituality to explain or believe why patients are not regaining their ‘normality’, as revealed by one young informant:

‘[*W*]hen I was still admitted at Rwanda military hospital my doctor told me that God might help and you get better or may not change and continue like that but with time you will adjust accept your situation.’ (Participant 3)

The comments generally offered a feeling of how spirituality is deep-rooted in the Rwandan community, where they say above all there is God. One informant described how he had developed suicidal thoughts instead of waiting for the last resort, which is God’s choice: ‘… I said to myself I should think of ending my life instead wait for what God will do for me …’ (Participant 4).

**Subcategory 1.6:** Complications were also seen by informants as a personal factor. Psychological and medical complications were common experiences such as depression, suicidal thoughts, feelings of worthlessness, and feelings of lack of confidence in accomplishing tasks, which otherwise they were capable of doing. In particular, some informants reported pressure ulcers and urinary and bowel incontinence. One informant discussed how incontinence strains his limited income, and he has multiple psychological and medical complications. He is a 35-year-old male nurse with a C4 injury:

‘I have a urinary catheter … I was using a condom catheter but because of PU I reverted to folic catheter … I used to have constipation, use laxatives but now I no longer however I do not control defecation, I use 9500frw on pampers per week. I have a lot of spasm, but I do not take any medications for them.’ (Participant 10)

#### Theme 2: Social relationships

This theme concerns all the relationships an individual has, including those with family members and those with neighbours or coworkers. The ‘dignity’ of the person with disability was strongly affected by attitudes and support from families, friends, and the society, which either facilitated or hindered the experience of inclusion in the community.

**Subcategory 2.1:** People with TSCI in this study who have supportive families, relatives, spouses, and siblings reported relatively better life experiences:

‘It’s a family that supports me especially my children who help me pay everything, my wife has been nursing me in the hospital and at home without them I would be badly off.’ (Participant 14)

Some informants reported declining family support with a decrease in the frequency of hospital visits when they were in acute care, and some even reported being abandoned when they had longer hospitalisations. A lengthy hospital stay is a barrier for these individuals since the patient cost of care, other than medical care, is essentially a family matter in Rwanda. One participant said, ‘initially many people visited me at the hospital but gradually people reduced in number’.

**Subcategory 2.2:** Those who received support from neighbours, friends, and initially workmates had a feeling of inclusion and being a useful member of the community.

For example:

‘My neighbour fabricated a standing and walking frame from wood, he did this at his expense, I feel he sees me as his neighbour but the rest do not check on me.’ (Participant 1)

In contrast, informants whose families or friends were not supportive either depended on hired care givers (if they could afford this) or were left to the mercy of social welfare services from the hospital and other caregivers.

One informant described the double burden of having been interactive and friendly but all of a sudden being abandoned by friends and family because of the TSCI:

‘… family members and friends will run away from you if they see your likely to need long term care they will exclude you. This is disturbing, the other thing is depending on a hired caregiver, he might decide to abandon you and you at risk of fall, developing PU …’ (Participant 10)

Participants report broken social ties because of the injury, as is reported by a 72-year-old male with tetraplegia, who had established himself as self-employed with planned retirement income and said:

‘… I am old but was productive now I am lying here sometimes in the rehab other time lying home useless … but even my children, friends used to come and we chat we share beer. My friends have lost a friend, company …’ (Participant 11).

Participants described how some members of the community use inappropriate language whilst referring to or relating with people with disabilities. The use of derogatory or inappropriate language may amount to discrimination. This affected individuals’ confidence and deterred people with TSCI from participating in social activities. One informant narrated how he faces barriers in implementing home exercise programmes because of neighbours’ attitudes, including inappropriate language:

‘My house is small, when I tried exercise in front of my house people crowded on me … he is crippled, he will never walk again, he is no longer a man, he is crazy … I felt that this environment cannot allow me to improve my health then I decided to shift to another place.’ (Participant 5)

#### Theme 3: Community-related factors

The community-related factors described by the individuals in this study included physical environment, community attitude, inappropriate mobility devices, and policies (i.e. health insurance and system inefficiencies).

**Subcategory 3.1:** Physical environment. The physical environment of the home was discussed as a barrier for some informants who would otherwise be independent in mobility and home activities. Often, the limitations were evident amongst individuals reporting stairs in the kitchen, doors, toilets, and backyards. Stairs were discussed as obstacles for otherwise independent people such as those with TSCI with low paraplegia who are using crutches and those using wheelchairs, ‘I entirely depend on wheelchair for mobility and it cannot go through the doors of this house, at the kitchen, toilet and bathroom entrance there are steps’ (Participant 7). Another informant who was improving from incomplete tetraplegia narrated how stairs at his house, his neighbours’ and at his workplace are a barrier:

‘I have issues with stairs at my house and even neighbours’ house almost all of them … I imagine when I am lucky and return to work, my work place has stairs everywhere.’ (Participant 9)

Another female informant told how her home compound nearly kept her totally indoors:

‘I would not get out of my house, the moment I would think of getting out of the house to some place I would think of mud, I would stay indoors only.’ (Participant 13)

The same informant stressed how the physical environment surrounding her community increased her cost of rehabilitation. Because of poor roads, she needs a private car to pick her up from her house to go to the rehabilitation centre. The poor roads increased the car hire charges: ‘going to the hospital was expensive, private taxi drivers would charge highly or refuse because the road was bad’ (Participant 13). These people were confident that their physical capacity allowed them to engage in basic activities like preparing their meals, accessing the toilet, making their rooms, and doing other home errands.

However, because of inaccessible home and community physical environments, they depended on their significant others for these activities.

Inaccessible physical environments in society and at home were one of the major barriers for the informants with regard to living a meaningful life, feeling included, and being independent.

**Subcategory 3.2:** The overall community attitude had an impact on these participants. The community physical environment and use of devaluating terminologies often lead to excluding people with disabilities from participating in real-life situations in their society. Devaluing attitudes towards persons with disabilities was evident, and it was thought to be partly because of old traditional belief systems held mainly within the older generations. However, the frustration participants felt with the attitude was reported to lessen with time after the acute period. Informants reported that, on average, it took 6 months living in the community until habituation and acceptance occurred. In the words of one participant:

‘When I put on my orthosis and it fails and I cannot walk I stop, cry and tell the caregiver to put me in a wheelchair and go back to the ward however it is less frequent now.’ (Participant 2)

**Subcategory 3.3:** Inappropriate mobility devices. Participants reported inappropriate mobility devices, especially wheelchairs and crutches. They pointed out that wheelchairs are either too small or too big and not terrain appropriate. Most of the wheelchairs are donated by charity organisations and constructed for use on different terrain. The informants’ compounds are mostly muddy or uneven, and this makes it difficult to use the donated wheelchairs that were made for a different terrain. ‘I was given a small wheelchair; people take measurements but return with inappropriate wheelchair … My first wheelchair was too small for me …’ (Participant 13).

**Subcategory 3.4:** Public policies. Informants described how existing public policies led to difficulties for those with TSCI because of reduced accessibility. For example, public building policies specifically regulated physical accessibility to public offices, banks, malls, courts of law, schools, and universities. The challenge was raised by informants seeking the aforementioned services and rights:

‘The other challenge is access due to physical environment barrier at offices, banks, I would send someone to tell the officer to come out and I tell him/her what I need at the bank. I have to sign a cheque for someone to draw money for me, yet I might need extra information on my account’, said an informant.’ (Participant 15)

**Subcategory 3.4.1:** System deficiencies in transportation. System deficiencies were cited primarily as shortages of a disability-friendly transport system. This was cited as a barrier for informants who are wheelchair users and who wanted to be mobile, work, and earn a living:

‘I can move around but it’s difficult, I cannot go back to my home area in the western part of Rwanda … I cannot use public transport; in Rwanda public means is buses or motorcycle yet I cannot use either of these.’ (Participant 15)

Another informant stressed the inconvenience faced in relation to transport:

‘… if I will go back home still using a wheelchair or people lifting me, I do not want it, I don’t believe that. If I can get orthosis with joints, it would be helpful otherwise boarding a bus, sitting in a bus is not convenient at all, actually not possible.’ (Participant 2)

**Subcategory 3.4.2:** Health insurance coverage. Health insurance was rated as highly helpful by almost all informants to access health care. It was rated as a facilitator by all the participants. Given the different health insurance plans in Rwanda, participants preferred public health insurance to private because public health insurance continued to pay their bills even when the beneficiary was no longer working. Private insurance stops with your work contract. However, a number of issues were raised in relation to health insurance; there are certain bills the insurance plans do not pay, ranging from wheelchairs to limitations in the number of sessions of rehabilitation. This is reflected in one of the discussions with an informant who was a complete paraplegic and had to depend on a wheelchair for mobility for the rest of his life: ‘there are things that community based health insurance (CBHI) does not cover like; wheelchair, orthoses, crutches, meals’ (Participant 4). Some insurance plans do not cover beyond a certain level of health care; tertiary and private hospitals versus only primary health care. As one of the informants stressed:

‘Yes, it is useful, its’ better than nothing because it supports in some health facilities but some it does not. like those hospitals at tertiary level with public and private affiliation or purely private, you need your own money or support.’ (Participant 4)

The burden of private insurance was described by one participant, a university graduate working with a private company, who was now a complete high paraplegic and no longer employed:

‘I was working and had an insurance plan, it helped me but my contract is ending at the end of the year. The challenge is that insurance allocates a certain amount in a year when you consume it before time then your foot the bill yourself 100%. This year I used a lot of money in rehab, when I had to buy an assistive device. My husband has a different insurance plan but does not cover private hospitals … when the employer pays to a better plan you benefit more but if the employer pays less, then the allocated quota monthly/annually is little then you suffer the hospital bills.’ (Participant 7)

#### Theme 4: Pre-injury context

Pre-injury context was raised by some participants who pointed out that one’s previous health, financial, and social status would affect post-injury wellbeing. One participant pointed out how her pre-injury status influenced her immediate effects of TSCI whilst in acute care:

‘I have realised that your childhood circumstances are likely to affect your adulthood, I had teenager pregnancy now an accident, it is as if it’s a punishment I am serving; why is it not getting finished because I had teenager pregnancy and now that is why I had an accident.’ (Participant 2)

Participants link negative incidents or events that happened in early life with their current situation that are because of totally different causes. If this type of event existed prior to the injury, it may greatly affect the psychological health of TSCI survivors. The situation could lead to self-isolation and consequently social exclusion, as described by one informant who had previously had a child at a young age:

‘My day was dominated by hiding in my bedroom I did not want anyone to see me. When friends would come to see me, I would tell my sisters to tell them that I am not around, it was stressful. People would collect around me and start wondering, they would say they have never seen someone who one time was walking and all of a sudden is bound in a wheelchair and wonder what will happen in the end.’ (Participant 2)

Informants reported previous domestic violence, either from parents or a spouse, as a big barrier to accepting their situation and meant not getting any family support. Domestic violence and suicidal thoughts looked like ‘twins’. They were often reported concurrently by the same people. A case in point is a 27-year-old participant who was a casual labourer and now has complete paraplegia. His parents were always fighting:

‘I was born in a poor and unpeaceful family … I wish I had died; I just do what I can if one feels sorry for me, he/she does the needful for me.’ (Participant 17)

#### Theme 5: Having a condition in common

Having a condition in common with others was raised by participants as a motivating factor. Having a common condition would demystify what happened to them, offer hope for the future, and promote acceptance. Informants stressed that they felt better when they were at the rehabilitation centre with colleagues who had similar conditions rather than at home, where there was either nobody who checked on them or he/she was the only one who could not stand and walk. Participants had a number of reasons why they preferred staying with colleagues with similar conditions, including staying away from people who knew them before injury, peer counselling, seeing the possibility for improvement, and hiding from unfriendly society:

‘Here I feel much better, I do not want to go back home, I am much better here, no one knows me here, I always want to stay where no one knows me. Every time I see people with similar condition.’ (Participant 2)

## Discussion

This study was undertaken to describe the experience of persons living with TSCI in the community in Rwanda. This is the first study of the experiences of individuals following TSCI living in the community in this country. The insights on their experiences living in the community after sustaining this life-altering event can contribute to planning for relevant programmes and interventions.

The major findings of this study concerned the informants’ personal resources in building coping strategies for living successfully following their injury. Source of income, family support, and having or sharing a common condition were important facilitators, whilst inaccessibility in the environment, devaluing attitudes, and pre-injury conditions were barriers. Functional limitations have been reported to limit participation in life situations (Gretschel, Visagie & Inglis 2017). This limits the possibility of getting involved in meaningful activity like employment and can lead to failure to earn any income, perform self-care activities, and engage in community social activities.

The results are reflected in other studies. One study in Ghana concerning the lived experience of people with SCI in a selected urban area reported pain, bladder and bowel problems, pressure ulcers, and neurological symptoms as limiting factors to their domestic duties, functioning and walking (Fuseini et al. 2019). Personal resources, such as income, have been reported by participants as an important facilitator in paying for their basic requirements.

However, most participants reported having to depend on their families and only a limited number on their prior savings. TSCI not only impoverishes people with the injury but also the family members who had been depending on the person and those that would shoulder the burden of caring for the person with SCI (Hossain et al. 2020). This clearly indicates how a shortage of income can limit people with TSCI to other paid services but also can cause poverty for families.

Unemployment is a major cause of limited personal resources, as reported by the study participants. This was also reported in a study from Bangladesh in which most of the people with SCI were housebound, unemployed, and reported a poor quality of life (Hossain et al. 2016). This might be an indication that providing mobility devices and skills training could help to change the situation of people with TSCI in low- and middle-income countries like Rwanda.

The findings of acceptance, self-confidence, and spirituality are of considerable importance after a devastating event, such as a TSCI. These factors were highlighted in a study conducted in Botswana (Löfvenmark et al. 2016). However, acceptance, self-confidence, and spirituality might mimic a picture of giving up on life because one is expected to restore his/her previous status, but it did not happen. Alternatively, low self-esteem might be because of limited patient information on his/her condition.

Spirituality is regarded as a solution when one is facing insurmountable problems in life. Such a situation is often a catalyst for people to search for an answer to their problems beyond the known scientific explanation.

Spirituality is well enshrined in the Rwandan community. Many Rwandans believe that God is the one who gives and takes away everything ranging from life, ability, possibility, and life (Vincent n.d.). The Rwandan expectation is that the elderly and individuals with disabilities are usually the responsibility of the family. This gives family members a feeling of responsibility for a member who has TSCI and is in need of support. However, on the other hand, it is stressful when the family does not have resources to support the person.

Social relationships are important in Rwandan society to the extent that when you do not receive frequent visits at your home, you are looked at as an unsocial, unfriendly, and unmanageable person. Losing old friends and failing to make new friends is something that affects one’s ‘dignity’. Interpersonal relationships create a sense of belonging in society, leading to inclusion.

An overall subliminal theme that coursed throughout the findings was that of loss of ‘dignity’.

Dignity in the Rwandan context means ‘Agaciro’, literally meaning value. Inappropriate language, physical environment, community attitude, and inappropriate devices were reported by participants in this study as barriers to inclusion in the community, and all led to the loss of dignity, and for some this had the further devastating consequence of thoughts of and attempts at suicide. None of these divisive factors are unique, having been previously reported in other studies conducted elsewhere (Babamohamadi et al. 2011; Joseph et al. 2016; Löfvenmark et al. 2016; Monden et al. 2014). Inappropriate language and community attitude can lead to stigmatisation and marginalisation, which in turn can affect dignity (Fuseini et al. 2019). A person can look down on him/herself as worthless and withdraw from social activities (Fuseini et al. 2019). These factors are likely to reduce one’s self-confidence and can lead to poor community participation and exclusion. Mobility devices were found to be inappropriate, as not all assistive devices are appropriately designed for all settings. The design should meet the users’ needs and be suitable in their physical, social, and cultural environment (Rohwerder 2018).

Assistive devices need to accommodate individual and environmental factors; otherwise, the device would not serve the purpose it is meant to serve. People with disabilities in African settings most of the time rely on donated devices from charity organisations, which are brought in from other settings and are thus often ineffective in the African settings (Burns & O’Connell 2012; Rohwerder 2018). Pre-injury conditions like low economic class families and domestic violence have also been cited as factors that also lead to reduced self-confidence and low self-esteem, hence causing a form of self-exclusion (Rohwerder 2018).

The participants in this study described how the construction of public buildings and the transport systems caused barriers to accessing services for people with TSCI. This system deficiency means people with TSCI are excluded from participating in their civic, religious, and social obligations.

Other studies have reported similar barriers to services for people with SCI (Akter et al., 2019; Republic of Rwanda – Ministry of Infrastructure 2012), raising the need for policy changes. Building codes and standards need to be enforced. The government needs to subsidise the importation and taxation of disability-friendly buses.

Health insurance policy has been reported as a facilitator for access to rehabilitation services. A supportive health insurance policy promotes health service access and has been cited in a systematic review carried out amongst low- and middle-income countries (Van Hees et al. 2019). Health insurances need to include all rehabilitation services and assistive devices on their paid list of services and items.

The WHO call for action 2030 clearly calls upon countries to address the significant unmet need for rehabilitation services and aims to integrate rehabilitation into universal health coverage and achieve the Sustainable Development Goals (WHO 2023). This call for action also resonates with the UN Commission on the Rights of Persons with Disability, which stipulates that access to rehabilitation and other services is a right (Degener 2017). This holds countries accountable to provide quality services, and Rwanda is a signatory. This means that Rwanda should do more in relation to improve the experience of people with SCI.

### Strengths

This study was the first to collect and report perspectives about living with TSCI in Rwanda. The results offer insight into the experiences and challenges these individuals face, and provide understanding about needed programme and policy changes in the country.

### Limitations

There are several limitations to consider in this work. The selection of the informants was purposive and may have introduced bias. During sampling, we deliberately enforced a variation in injury level, rural and urban settings, severity of injury, and gender balance. However, this purposive sampling will not cover all situations. The reporting of the experiences was limited by what participants chose to share. There could be additional insights they selected not to tell the researcher about.

## Conclusion

The challenges experienced by people with TSCI can be influenced by many diverse factors ranging from personal to environmental factors and employment to policy issues. Personal resources and income affect every layer of living conditions of a person. This study highlighted many personal, social, and community factors that facilitate or hinder inclusion of people with TSCI as they recover from their injury and engage in the community. Key facilitators identified were strong personal resources, a supportive family, health insurance coverage, and peer counselling. Barriers include inaccessibility to public buildings and transport, inappropriate assistive devices, inappropriate language, and pre-injury conditions. It is the responsibility of the government and communities to build a facilitating environment; the results show poor reintegration, which is contrary to the international conventions provisions, as earlier pointed out, of which the country is a signatory. People with TSCI should have a satisfactory level of community reintegration and the ability to live fulfilling and participating lives. Future research should explore reasons why governments have been slow on implementing strategies to facilitate persons with disabilities to fully reintegrate back into their community.

## References

[CIT0001] Akter, F., Islam, S., Haque, O., Hossain, A., Hossain, K.M.A., Imran, M. et al., 2019, ‘Barriers for individuals with spinal cord injury during community reintegration: A qualitative study’, *International Journal of Physical Medicine and Rehabilitation* 7(3), 1–7.

[CIT0002] Almalki, S., 2016, ‘Integrating quantitative and qualitative data in mixed methods research – Challenges and benefits’, *Journal of Education and Learning* 5(3), 288. 10.5539/jel.v5n3p288

[CIT0003] Babamohamadi, H., Negarandeh, R. & Dehghan-Nayeri, N., 2011, ‘Barriers to and facilitators of coping with spinal cord injury for Iranian patients: A qualitative study’, *Nursing and Health Sciences* 13(2), 207–215. 10.1111/j.1442-2018.2011.00602.x 21595815

[CIT0004] Burns, A.S. & O’Connell, C., 2012, ‘The challenge of spinal cord injury care in the developing world’, *Journal of Spinal Cord Medicine* 35(1), 3–8. 10.1179/2045772311Y.0000000043 22330185 PMC3240914

[CIT0005] Cripps, R.A., Lee, B.B., Wing, P., Weerts, E., Mackay, J. & Brown, D., 2011, ‘A global map for traumatic spinal cord injury epidemiology: Towards a living data repository for injury prevention’, *Spinal Cord* 49(4), 493–501. 10.1038/sc.2010.146 21102572

[CIT0006] Degener, T., 2017, ‘10 years of convention on the rights of persons with disabilities’, *Netherlands Quarterly of Human Rights* 35(3), 152–157. 10.1177/0924051917722294

[CIT0007] Donovan, W.H., 2007, ‘Donald Munro lecture: Spinal cord injury – Past, present, and future’, *The Journal of Spinal Cord Medicine* 30(2), 85–100. 10.1073/pnas.89.12.5670 17591221 PMC2031949

[CIT0008] Fuseini, A.G., Aniteye, P. & Alhassan, A., 2019, ‘Beyond the diagnosis: Lived experiences of persons with spinal cord injury in a selected town in Ghana’, *Neurology Research International* 2019, 10. 10.1155/2019/9695740 PMC6354163 30792925

[CIT0009] Gretschel, D., Visagie, S. & Inglis, G., 2017, ‘Community integration of adults with disabilities post discharge from an in-patient rehabilitation unit in the Western Cape’, *South African Journal of Physiotherapy* 73(1), 1–7. 10.4102/sajp.v73i1.361 PMC6093139 30135906

[CIT0010] Hisham, H., Rosley, H., Manaf, H. & Justine, M., 2022, ‘Barriers and facilitators to physical activity and exercise among individuals with spinal cord injury: A systematic review’, *Malaysian Journal of Medicine and Health Sciences* 18(8), 365–373. 10.47836/mjmhs18.8.46

[CIT0011] Hossain, M.S., Harvey, L.A., Islam, M.S., Rahman, M.A., Liu, H. & Herbert, R.D., 2020, ‘Loss of work-related income impoverishes people with SCI and their families in Bangladesh’, *Spinal Cord* 58(4), 423–429. 10.1038/s41393-019-0382-1 31772346 PMC7138756

[CIT0012] Hossain, M.S., Rahman, M.A., Bowden, J.L., Quadir, M.M., Herbert, R.D. & Harvey, L.A., 2016, ‘Psychological and socioeconomic status, complications and quality of life in people with spinal cord injuries after discharge from hospital in Bangladesh: A cohort study’, *Spinal Cord* 54(6), 483–489. 10.1038/sc.2015.179 26458967

[CIT0013] Joseph, C., Wahman, K., Phillips, J. & Wikmar, L.N., 2016, ‘Client perspectives on reclaiming participation after a traumatic spinal cord injury in South Africa’, *Physical Therapy* 96(9), 1372–1380. 10.2522/ptj.20150258 27081200

[CIT0014] Kanyoni, M., Nilsson-Wikmar, L., Phillips, J. & Tumusiime, D.K., 2023, ‘Quality of life after traumatic spinal cord injury in Rwanda, the impact of personal and contextual factors: A follow-up exploratory study’, *Rwanda Journal of Medicine and Health Sciences* 6(3), 326–334. 10.4314/rjmhs.v6i3.6 40568644 PMC12110498

[CIT0015] Kanyoni, M., Wikmar, L.N., Philips, J., Joseph, C. & Tumusiime, D.K., 2024a, ‘Incidence and etiology of traumatic spinal cord injury in Rwanda: A prospective population-based study’, *Frontiers in Neurology* 15, 1373893. 10.3389/fneur.2024.1373893 39233676 PMC11371736

[CIT0016] Kanyoni, M., Wikmar, L.N., Philips, J. & Tumusiime, D.K., 2024b, ‘Psychosocial reintegration post-traumatic spinal cord injury in Rwanda: An exploratory study’, *South African Journal in Physiotherapy* 80(1), 1996. 10.4102/sajp.v80i1.1996 PMC10913185 38445219

[CIT0017] Löfvenmark, I., Hasselberg, M., Nilsson Wikmar, L., Hultling, C. & Norrbrink, C., 2017, ‘Outcomes after acute traumatic spinal cord injury in Botswana: From admission to discharge’, *Spinal Cord* 55(2), 208–212. 10.1038/sc.2016.122 27527239

[CIT0018] Löfvenmark, I., Norrbrink, C., Wikmar, L.N. & Löfgren, M., 2016, ‘“The moment I leave my home – There will be massive challenges”: Experiences of living with a spinal cord injury in Botswana’, *Disability and Rehabilitation* 38(15), 1483–1492. 10.3109/09638288.2015.1106596 26694314

[CIT0019] Monden, K.R., Trost, Z., Catalano, D., Garner, A.N., Symcox, J., Driver, S. et al., 2014, ‘Resilience following spinal cord injury: A phenomenological view’, *Spinal Cord* 52(3), 197–201. 10.1038/sc.2013.159 24418959

[CIT0020] National Institute of Statistics of Rwanda, and Ministry of Finance and Economic Planning, 2014, *Rwanda fourth population and housing census. Final results: Publication* tables, National Institute of Statistics of Rwanda, Kigali.

[CIT0021] Nowell, L.S., Norris, J.M., White, D.E. & Moules, N.J., 2017, ‘Thematic analysis: Striving to meet the trustworthiness criteria’, *International Journal of Qualitative Methods* 16(1), 1–13. 10.1177/1609406917733847

[CIT0022] Republic of Rwanda – Ministry of Infrastructure, 2012, *Republic of Rwanda Ministry of Infrastructure Public Transport Policy and Strategy*.

[CIT0023] Rohwerder, B., 2018, *Assistive technologies in developing countries*, Helpdesk Report K4D: Knowledge, Evidence, and Learning for Development, Institute of Development Studies, London.

[CIT0024] Shenton, A.K., 2004, ‘Strategies for ensuring trustworthiness in qualitative research projects’, *Education for Information* 22(2), 63–75. 10.3233/EFI-2004-22201

[CIT0025] Van Hees, S.G.M., O’Fallon, T., Hofker, M., Dekker, M., Polack, S., Banks, L.M. et al., 2019, ‘Leaving no one behind? Social inclusion of health insurance in low- and middle-income countries: A systematic review’, *International Journal for Equity in Health* 18(1), 134. 10.1186/s12939-019-1040-0 31462303 PMC6714392

[CIT0026] Vincent, M., n.d., *Republic of Rwanda: Rwandan cultural values in national development*, pp. 1–74, National Commission for Unity and Reconciliation, Kigali.

[CIT0027] Wong, M.M.Y., Seliman, M., Loh, E., Mehta, S. & Wolfe, D.L., 2022, ‘Experiences of individuals living with spinal cord injuries (SCI) and acquired brain injuries (ABI) during the COVID-19 pandemic’, *Disabilities* 2(4), 750–763. 10.3390/disabilities2040052

[CIT0028] World Health Organization, 2003, *International perspectives on spinal cord injury*, p. 250, The National Association of Resident Doctors of Nigeria, WHO, Malta. 10.1007/978-1-4899-1028-8_18

[CIT0029] World Health Organization, 2013, ‘International perspectives on spinal cord injury’, *The Lancet* 1–247.

[CIT0030] World Health Organization, 2023, *Meeting resolutions: The participants of the meeting Rehabilitation 2030 acknowledge the following*, viewed July 2022, from https://www.who.int/docs/default-source/documents/health-topics/rehabilitation/callforaction2.pdf?sfvrsn=50299fc6_2.

